# Significance of elevated serum and hepatic NOD-like receptor pyrin domain containing 3 (NLRP3) in hepatitis C virus-related liver disease

**DOI:** 10.1038/s41598-022-22022-5

**Published:** 2022-11-14

**Authors:** Hoda El Aggan, Sabah Mahmoud, Nevine El Deeb, Islam Eleishi, Assem El-Shendidi

**Affiliations:** 1 Department of Internal Medicine (Hepatobiliary Unit), Faculty of Medicine, Alexandria University, Alexandria, Egypt; 2 Department of Medical Biochemistry, Faculty of Medicine, Alexandria University, Alexandria, Egypt; 3 Department of Pathology, Faculty of Medicine, Alexandria University, Alexandria, Egypt

**Keywords:** Biochemistry, Gastroenterology

## Abstract

NOD-like receptor pyrin domain containing 3 (NLRP3) is a microbial and danger signal sensor that acts as a regulator of inflammation via activation of Caspase-1 (CASP1) and has been identified as a major contributor to human liver diseases. The present study was conducted to investigate the association between NLRP3 and the progression of hepatitis C virus (HCV)-related liver disease. Serum NLRP3 levels were analyzed in 49 patients with chronic HCV infection and 18 healthy controls and liver tissues from 34 patients were examined to assess the protein expression of NLRP3 and its activation marker CASP1 using immunohistochemical staining. The results showed that the median serum NLRP3 levels was significantly higher in HCV-infected patients compared with healthy controls (1040 pg/ml vs 695 pg/ml respectively, *P* < 0.001) and were positively correlated with hepatic NLRP3 and CASP1 expression (r = 0.749, *P* < 0.001 and r = 0.557, *P* = 0.001 respectively). The NLRP3 levels in serum and the liver significantly increased with worsening liver pathology and showed positive correlations with serum aminotransferases levels, HCV viremia, and albumin-bilirubin score (*P* < 0.05). The receiver operating characteristic curve analysis revealed a high diagnostic performance of serum NLRP3 in determining the extent of liver necroinflammation, fibrosis, and steatosis (area under the curve = 0.951, 0.971, and 0.917 respectively, *P* < 0.001). In conclusion, NLRP3 plays an important role in liver disease progression during HCV infection via CASP1 activation and might be a promising therapeutic target. Serum NLRP3 could be an additional biomarker for liver inflammation and fibrosis.

## Introduction

Hepatitis C virus (HCV), a single-stranded RNA member of the *Flaviviridae* family, is a major cause of chronic liver disease leading to chronic hepatitis, progressive fibrosis, and eventually cirrhosis with an increased risk for the development of hepatocellular carcinoma^1^. Chronic HCV infection evolves a chronic inflammatory response in the liver due to the induction of pro-inflammatory cytokines upon recognition of pathogen-associated molecular patterns (PAMPs) and host-derived damage-associated molecular patterns (DAMPs) by pattern recognition receptors (PRRs) of the innate immune system^2^. Sustained inflammatory stimuli promote hepatic stellate cells (HSCs) activation with the accumulation of extracellular matrix resulting in hepatic fibrosis^3^. Also, the inflammatory response contributes to the accumulation of lipid droplets in the hepatocytes and the evolution of hepatic steatosis during HCV infection^4^. Moreover, persistent, ongoing inflammation may increase the risk of long-term liver-related complications in patients with advanced chronic liver disease after direct-acting antiviral therapy^5^. Defining the processes that promote hepatic inflammation by HCV is paramount for establishing approaches to minimize liver disease progression^6^.

Nucleotide-binding and oligomerization domain (NOD)-like receptors (NLR) are cytosolic microbial and danger signal sensors/PRRs that act as key regulators of inflammation^7^. Among the NLR family members, the NLR pyrin domain-containing 3 (NLRP3) is currently the best characterized and most extensively studied because of its role in many viral and inflammatory diseases^8^. Upon recognition of PAMPs or DAMPs, NLRP3 forms a multi-protein complex known as the inflammasome leading to proteolytic cleavage of procaspase-1 into active Caspase-1 (CASP1), which in turn mediates the maturation of the pro-inflammatory cytokines interleukin (IL)-1β and IL-18 and triggers an inflammatory form of cell death known as pyroptosis^9,10^. NLRP3 up-regulation requires a priming signal such as Toll-like receptors (TLRs), to activate nuclear factor kappa-B (NF-κB) resulting in its binding to the NLRP3 promoter. Once primed, NLRP3 can respond to cellular stimuli leading to the assembly of NLRP3 inflammasome^11^.

Many RNA viruses can induce NLRP3 transcription by interacting with TLRs and promote NLRP3 inflammasome activation to influence the inflammatory response^12^. NLRP3 plays a central role in the innate immune responses to viral infections through CASP1-mediated secretion of pro-inflammatory cytokines and induction of pyroptotic cell death leading to lysis of virus-infected cells^13^. However, dysregulated inflammasome activation may result in a rapid and broad inflammatory response leading to virus persistence^12^.

In the liver, NLRP3 is expressed in both hepatocytes and non-parenchymal liver cells and can sense and respond to various microbial particles that reach the liver by portal circulation^14^. However, while NLRP3 is essential for defense against pathogens and danger signals, excessive activation of the NLRP3 inflammasome can trigger hepatocyte damage, immune cell activation, and amplification of liver inflammation and fibrosis^15,16^. Also, NLRP3 is involved in the pathogenesis of hepatic steatosis^17^. NLRP3 activation has been identified as a major contributor to human liver diseases^17–19^ and experimental models of liver injury^20–24^. However, the role of NLRP3 in HCV-related liver disease has not been fully elucidated.

Therefore, the present work was conducted to investigate the association between NLRP3 and the progression of HCV-related liver disease by studying serum levels and hepatic expression of NLRP3 in patients with chronic HCV infection and their relation to the severity of liver injury and also, to explore whether serum NLRP3 could be a potential biomarker for HCV-related liver pathology.

## Materials and methods

### Study population

The present work is a case–control study and included a total of 49 treatment-naïve patients with HCV-related liver disease, who were referred between January 2018 to June 2019 to the Hepatobiliary Unit, Department of Internal Medicine, at the Main Alexandria University Hospital, Alexandria, Egypt. The patients were 35 males and 14 females and their ages ranged between 23 and 55 years (mean ± s.d. = 40.07 ± 7.34 years). All patients had seropositivity for HCV antibody and detectable serum HCV RNA and were selected from 73 HCV-infected patients after exclusion of hepatitis B virus infection; alcohol consumption; other causes of chronic liver disease; diabetes mellitus; hyperlipidemia; autoimmune diseases; other infectious or inflammatory diseases; malignancy; cardiac, respiratory, or renal disease; and previous intake of anti-viral treatment or anti-inflammatory drugs. Also, 18 age- and sex-matched healthy subjects with no evidence of liver disease were included in the study. They were 14 males and 4 females and their ages ranged between 25 and 56 years (mean ± s.d. = 42.61 ± 8.20 years). The study was conducted according to the Code of Ethics of the World Medical Association (Declaration of Helsinki) and was approved by the Institutional Review Board/Ethics Review Committee of the Faculty of Medicine (Approval number: 0105325). Informed consent was obtained from all participants in the study.

### Clinical and laboratory data

All patients were evaluated clinically focusing on age, sex, and manifestations of chronic liver disease. Abdominal ultrasonography was performed to assess liver size and echo pattern and the presence of cirrhosis, splenomegaly, and ascites. Laboratory investigations included complete blood picture, liver test profile [serum aspartate aminotransferase (AST), alanine aminotransferase (ALT), gamma-glutamyl transpeptidase (GGT), albumin, and bilirubin, prothrombin time (PT), and international normalized ratio (INR)], serum creatinine, serum sodium (Na), and serum high-sensitivity C-reactive protein (hsCRP), a marker of systemic inflammation. HCV RNA levels in serum were quantified using reverse transcription real-time polymerase chain reaction (COBAS Ampliprep/COBAS TaqMan; Roche Diagnostic Systems AQ5, USA).

### Assessment of the severity of liver dysfunction

The severity of liver dysfunction was assessed according to the Child–Pugh classification^25^, the Model for End-Stage Liver Disease Sodium (MELDNa) score^26^, and the albumin-bilirubin (ALBI) score in patients with cirrhosis^27^. The Child–Pugh classification depends on 5 parameters: encephalopathy, ascites, albumin, total bilirubin, and PT^25^. The MELDNa score was calculated according to the following equation:

$$MELDNa \, = \, [MELD \, + \, (140 \, {-} \, Na\left( {mmol/L} \right)) \, {-} \, (0.025 \, \times \, MELD \, \times \, (140 \, {-} \, Na \, (mmol/L))]$$

where the serum Na concentration is bound between 125 and 140 mmol/L^26^. The ALBI score was calculated as follows:

$$ALBI \, score \, = \, [(Log_{10}\, total \, bilirubin \, \left( {\mu mol/l} \right) \, \times \, 0.66) \, + \, \left( {albumin \, \left( {g/l} \right) \, \times \, - 0.085} \right)]$$

and was graded into grade 1 (score ≤ −2.60), grade 2 (score > −2.60 to ≤ −1.39) and grade 3 (score > −1.39)^27^.

### Noninvasive liver fibrosis scores

The noninvasive liver fibrosis scores including AST to platelet ratio index (APRI) and Fibrosis-4 index (FIB-4) were calculated using the following equations:^28^.

$$APRI \, = \left[ {AST \, \left( {U/L} \right) \, / \, upper \, limit \, of \, normal \, AST \, values \, \left( {U/L} \right)} \right] \, / \, \left[ {Platelets \, \left( {10^{9} /L} \right)} \right] \, x \, 100$$

$$FIB - 4 \, = \, \left[ {age \, \times \, AST \, \left( {U/L} \right)} \right] \, / \, [Platelet \, \left( {10^{9} /L} \right) \, x \, \surd ALT \, (U/L)]$$

### Measurement of serum NLRP3 levels by ELISA

Serum NLRP3 levels were measured in duplicate for each sample using a commercial sandwich-enzyme-linked immunosorbent assay (ELISA) kit (Human NACHT, LRR, and pyrin domains-containing protein 3 (NLRP3) ELISA Kit, INOVA, Beijing, China) according to the manufacturer’s instructions. The assay range was between 56–4000 pg/ml and the sensitivity of the kit was 12 pg/ml. The intra-assay and inter-assay coefficients of variation were < 10% and < 12% respectively. Briefly, 10 μl diluted sample (dilution factor was 5) and standards were added to the appropriate Microelisa stripplate wells pre-coated with an antibody specific to NLRP3 and incubated for 30 min at 37 ºC. Then, 50 μl Horseradish Peroxidase-conjugate reagent was added to each well except the blank control well and incubated for 30 min at 37 °C. After free components were washed away, TMB (3,3',5,5'-tetramethylbenzidine) substrate solution was added to each well and then, 50 μl stop solution was added to terminate the reaction. The color in the well changed from blue to yellow. The absorbance of the samples was measured spectrophotometrically at a wavelength of 450 nm and its value was proportional to the concentration of NLRP3. The concentration of NLRP3 in sample was interpolated using the standard curve constructed by the ELISA reader model. The original concentration was calculated by multiplying the dilution factor.

### Histopathological examination

Core liver biopsies were collected from 34 patients with HCV-related liver disease and were fixed in 10% formalin solution, embedded in paraffin, sectioned (5 μm), and subsequently stained with hematoxylin–eosin and trichrome stains. Liver specimens were examined by an experienced pathologist who had been blinded to the clinical results. Histological activity grade (A0-A3) and fibrosis stage (F0-F4) were assessed according to the METAVIR scoring system. Cirrhosis was designated as fibrosis stage F4^29^. The grade of steatosis was determined as the percentage of lipid droplet-containing hepatocytes according to Brunt et al.^30^ as follows: grade 0: 0–2% (none), grade 1: 3–29% (mild), grade 2: 30–60% (moderate), and grade 3: > 60% (severe).

### Immunohistochemical staining

Immunohistochemical staining of NLRP3 and its activation marker CASP1 in the liver was performed. The streptavidin–biotin-peroxidase method was applied using the UltraVision LP detection system (Thermo Fisher Scientific, Fremont, CA, USA). Liver sections were deparaffinized and incubated with the following primary antibodies at 4 °C overnight in a humid chamber: *anti-NLRP3 antibody* (CSB-PA015871LA01HU)—rabbit polyclonal antibody at a dilution 1:20 (CUSABIO, Wuhan, Hubei Province, China); and *anti-CASP1* (*cleaved*) *antibody* (MBS301007)—rabbit polyclonal antibody at a dilution 1:50 (MyBioSource, San Diego, CA, USA). Slides were then incubated with biotinylated goat anti-polyvalent (linking reagent), followed by peroxidase-conjugated streptavidin, each for 20 min at room temperature. Tissue sections were washed with phosphate-buffered saline for 5 min after each step. A brown color reaction was developed by using 3–3 diaminobenzidine tetrahydrochloride mixture for 10 min. The slides were finally dehydrated, counterstained with hematoxylin, and mounted. Negative control sections (where the primary antibody has been omitted), were included in each run.

### Interpretation of immunohistochemical analysis

Cells with brownish granules in the cytoplasm were considered as positive cells and cells with no coloration or consistent with the background color were considered as negative cells. The *staining intensity score* for NLRP3 was classified as: 0 (negative), 1 (weak), 2 (moderate) and 3 (strong), and the *staining proportion score* was classified as: 0 (0%), 1 (1–25%), 2 (26–50%), 3 (51–75%) and 4 (76–100%). The final staining score for NLRP3 was calculated by multiplying the *staining intensity score* by the *staining proportion score* and accordingly, patients were divided into two groups: low expression group (score 0–6) and high expression group (score 7–12)^31^. The staining intensity score for CASP1 was graded semi-quantitatively as score 0 (negative), score 1 (weak), score 2 (moderate), and score 3 (strong)^32^.

### Statistical analysis

Statistical analysis was performed using SPSS 22.0 software (Armonk, NY: IBM Corp.). The normality of data was determined by Kolmogorov–Smirnov and Shapiro–Wilk tests. Continuous variables were expressed as mean ± standard deviation (s.d.) if normally-distributed and as median (interquartile range (IQR)) if non-normally distributed. Categorical variables were presented as numbers (percentages). The study groups were compared using the Student’s *t* test or the Mood’s median test as appropriate for continuous variables, and the Fisher’s Exact test (*FET*) with Monte Carlo corrected significance for categorical variables. Correlations between continuous variables were analyzed by using Spearman’s rank test. The receiver operating characteristic (ROC) curve was plotted to determine the sensitivity, specificity, area under the curve (AUC), 95% confidence interval, and the best cut-off value of serum NLRP3 and other tests in determining the extent of liver pathology. The positive predictive value (PPV), negative predictive value (NPV), and accuracy were calculated at the same cut-off value. The comparison between the AUC of two ROC curves was performed using DeLong’s test (MedCalc version 19.3, MedCalc software Ltd., Ostend, Belgium). Statistical significance was assessed at *P* ≤ 0.05 and all calculated *P* values were two-tailed.

## Results

### Characteristics of subjects

Table 1 summarizes the clinical, laboratory, and histopathological data of patients with HCV-related liver disease and healthy controls. The age and sex showed no statistically significant differences between studied groups (*P* = 0.830 and *P* = 0.760 respectively). Serum levels of AST, ALT, GGT, total bilirubin, and hsCRP, PT, and INR showed significant increases, while platelet count and serum levels of albumin and Na showed significant decreases in patients with HCV-related liver disease compared with healthy controls (*P* < 0.05). Serum HCV-RNA levels ranged between 7.7 and 7400 × 10^3^ IU/ml [median (IQR): 900 (1833.5)]. In patients with cirrhosis, the Child–Pugh score ranged between 5 and 8 (mean ± s.d.: 5.86 ± 1.06, class A in 15 (71.4%) patients and class B in 6 (28.6%) patients), the MELDNa score ranged between 6 and 19 (mean ± s.d.: 12.52 ± 3.54), and the ALBI score ranged between −3.33 and −1.25 [mean ± s.d.: −2.51 ± 0.49]. The APRI and FIB-4 ranged between 0.32 and 4.56 [median (IQR): 1.31 (1.47)] and 0.87–4.49 [median (IQR): 1.61 (1.18)] respectively. According to METAVIR scoring system, the histological activity grade was classified as mild activity (A1) in 5 (14.7%) patients, moderate activity (A2) in 16 (47.1%) patients, and severe activity (A3) in 16 (47.1%) patients while fibrosis stage was F1 in 5 (14.7%) patients, F2 in 12 (35.3%) patients, F3 in 2 (5.9%) patients, and F4 (cirrhosis) in 15 (44.1%) patients. Steatosis was graded as grade 0 in 15 (44.1%) patients, grade 1 in 8 (23.5%) patients, grade 2 in 9 (26.5%) patients, and grade 3 in 2 (5.9%) patients.

**Table 1:** Clinical, laboratory, and histopathological data of patients with hepatitis C virus (HCV)-related liver disease and healthy controls.

Variables	HCV-related liver disease (n = 49)	Healthy controls (n = 18)	*P*-value*
Age (years)	42.16 ± 7.32	42.61 ± 8.20	0.830^a^
Sex			
Male, *n* (%)	35 (71.4)	14 (77.8)	0.760^b^
Female, *n* (%)	14 (28.6)	4 (22.2)	
Hemoglobin (g/dl)	13.58 ± 1.25	14.16 ± 0.81	0.074^a^
Platelets (× 10^3^/mm^3^)	189.08 ± 51.43^†^	311.11 ± 46.86	< 0.001^a^
AST (U/L)	70.82 ± 44.17	19.44 ± 4.00	< 0.001^a^
ALT (U/L)	83.57 ± 53.19	18.11 ± 3.55	< 0.001^a^
GGT (U/L)	49.59 ± 27.68	24.21 ± 5.46	< 0.001^a^
Albumin (g/dl)	3.88 ± 0.48	4.56 ± 0.37	< 0.001^a^
Total bilirubin (mg/dl)	1.02 ± 0.52	0.65 ± 0.14	0.005^a^
PT (seconds)	12.11 ± 1.15	11.27 ± 0.58	0.004^a^
INR	1.12 ± 0.09	1.07 ± 0.08	0.010^a^
HCV RNA (× 10^3^ IU/ml), median (IQR)	900 (1,833.5)	–	
Creatinine (mg/ml)	0.74 ± 0.22	0.67 ± 0.18	0.239^a^
Sodium (mmol/L)	137.67 ± 3.15	140.28 ± 2.14	0.003^a^
hsCRP (mg/dl)	0.60 ± 0.30	0.31 ± 0.04	< 0.001^a^
Child–Pugh, (n = 21)			
Score	5.86 ± 1.06	–	
Class A, *n* (%)	15 (71.4)	–	
Class B, *n* (%)	6 (28.6)	–	
MELDNa score, (n = 21)	12.52 ± 3.54	–	
ALBI score, (*n* = 21)	-2.51 ± 0.49	–	
Grade 1, *n* (%)	3 (14.3)	–	
Grade 2, *n* (%)	17 (81.0)	–	
Grade 3, *n* (%)	1 (4.8)	–	
APRI, median (IQR)	1.31 (1.47)	–	
FIB-4, median (IQR)	1.61 (1.18)	–	
Histological activity grade (n = 34)			
A1, *n* (%)	5 (14.7)	–	
A2, *n* (%)	16 (47.1)	–	
A3, *n* (%)	13 (38.2)	–	
Fibrosis stage (n = 34)			
F1, *n* (%)	5 (14.7)	–	
F2, *n* (%)	12 (35.3)	–	
F3, *n* (%)	2 (5.9)	–	
F4 (cirrhosis), *n* (%)	15 (44.1)	–	
Steatosis grade (n = 34)			
Grade 0, *n* (%)	15 (44.1)	–	
Grade 1, *n* (%)	8 (23.5)	–	
Grade 2, *n* (%)	9 (26.5)	–	
Grade 3, *n* (%)	2 (5.9)	–	

AST, Aspartate aminotransferase; ALT, Alanine aminotransferase; GGT, Gamma-glutamyl transpeptidase; PT, Prothrombin time; INR, International normalized ratio; IQR, Interquartile; hsCRP, High-sensitivity C-reactive protein; MELDNa; Model for End-Stage Liver Disease Sodium; ALBI, Albumin-bilirubin; APRI, Aspartate aminotransferase to platelet ratio index; FIB-4, Fibrosis-4 index.

^a^Student’s t test; ^b^Fisher’s Exact test with Monte Carlo corrected significance.

*Statistically significant at *P* ≤ 0.05.

### Serum NLRP3 levels significantly increased in patients with hepatitis C virus-related liver disease

**Figure 1:**
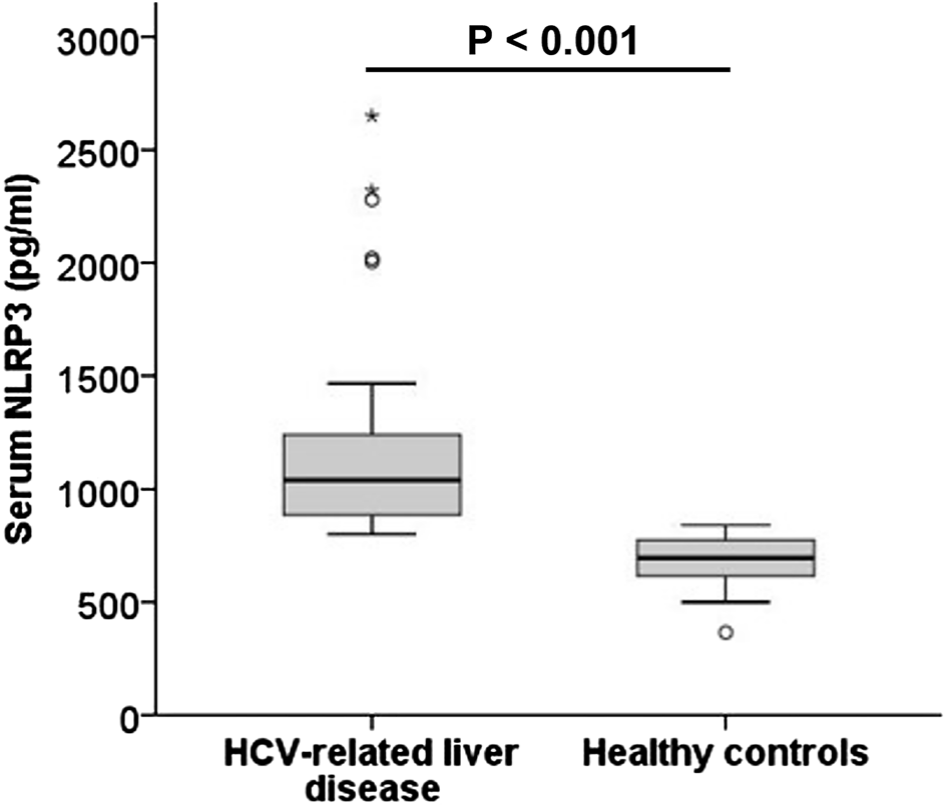
Serum nucleotide-binding and oligomerization domain-like receptor pyrin-domain-containing 3 (NLRP3) level (pg/ml) in patients with HCV-related liver disease and healthy controls [Data are expressed as median (interquartile range), 1040 (395) pg/ml vs 695 (183) pg/ml respectively, χ^2^ = 23.888, *P* < 0.001, the Mood’s median test].

Serum NLRP3 level ranged between 800 and 2,650 pg/ml in patients with HCV-related liver disease and between 365 and 840 pg/ml in healthy controls. The serum NLRP3 levels were significantly higher in patients with HCV-related liver disease than in healthy controls [median (IQR): 1040 (395) pg/ml vs 695 (183) pg/ml respectively, χ^2^ = 23.888, *P* < 0.001, the Mood’s median test] (Fig. 1).

### Hepatic NLRP3 and Caspase-1 expression by immunohistochemistry

**Figure 2:**
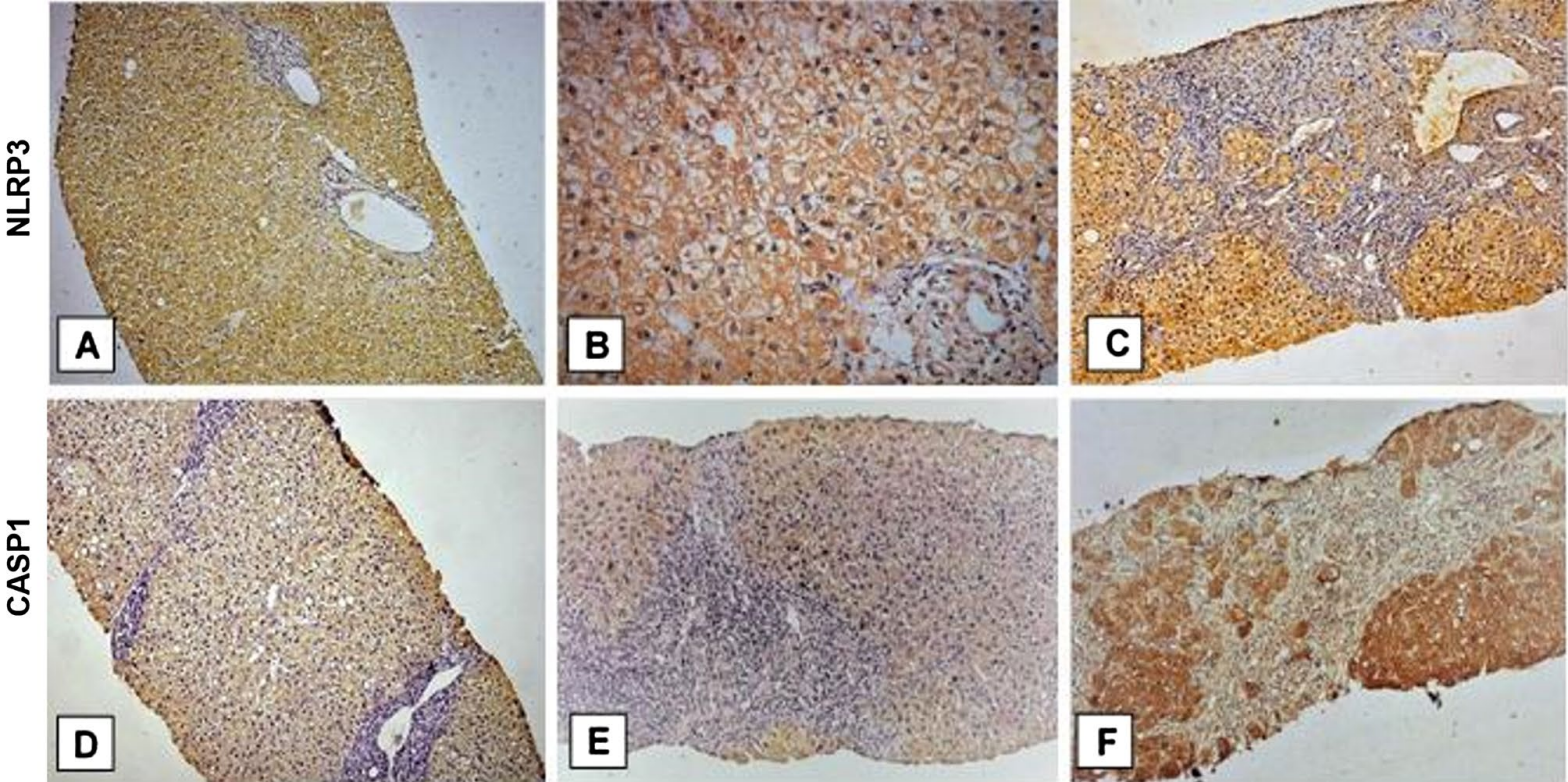
Immunohistochemical staining of liver tissues from patients with hepatitis C virus-related liver disease. (**A**) METAVIR stage F1 with mild activity (A1), and no steatosis. Moderate brown positive NLRP3 (nucleotide-binding and oligomerization domain-like receptor pyrin-domain-containing 3) staining of hepatocytes is seen (staining score 4, low expression) (anti-NLRP3 × 200); (**B**) High power view demonstrating brown cytoplasmic NLRP3 expression in hepatocytes (anti-NLRP3 × 400); (**C**) METAVIR stage F4 (cirrhosis) with moderate activity (A2), and steatosis. Strong NLRP3 expression is seen (staining score 12, high expression) (anti-NLRP3 × 200); (**D**) METAVIR stage F2, with moderate activity (A2). Weak brown positive Caspase-1 (CASP1) staining of hepatocytes is observed (staining score 1) (anti-CASP1 × 200). (**E**) METAVIR stage F3 with moderate activity (A2), and no steatosis. Moderate brown positive CASP1 staining of hepatocytes is seen (staining score 2) (anti-CASP1 × 200); and (**F**) METAVIR stage F4 (cirrhosis). Strong brown positive CASP1 staining of hepatocytes (staining score 3) (anti-CASP1 × 200).

**Figure 3:**
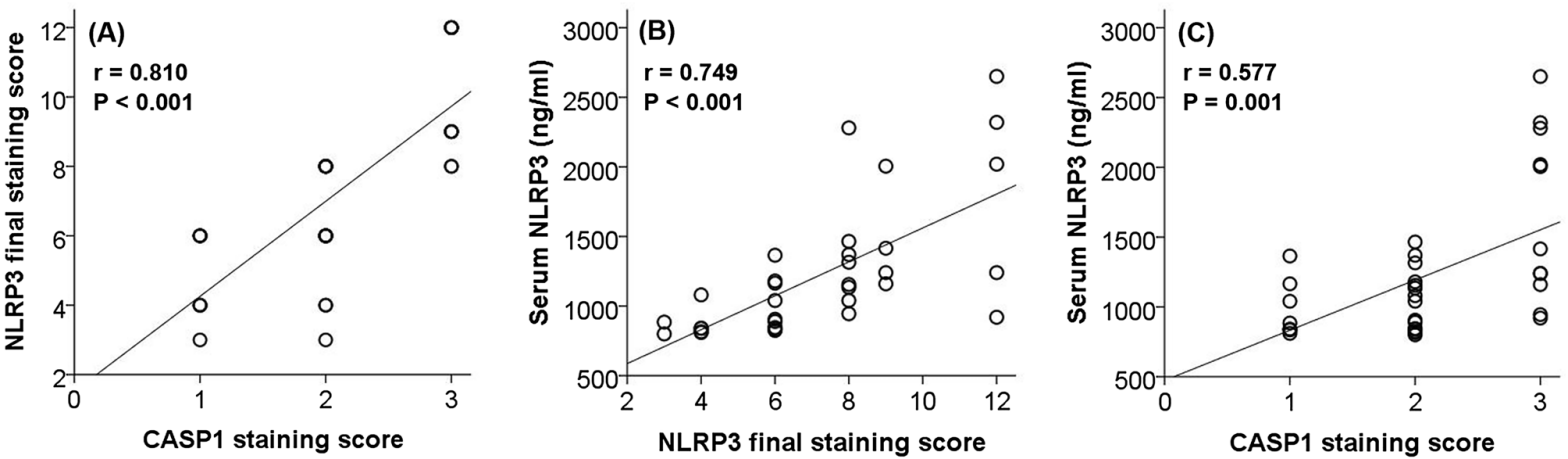
Statistical correlations between (**A**) hepatic nucleotide-binding and oligomerization domain-like receptor pyrin-domain-containing 3 (NLRP3) and Caspase-1 (CASP1) staining scores (r = 0.810, *P* < 0.001), (**B**) serum NLRP3 levels (pg/ml) and hepatic NLRP3 final staining score (r = 0.749, *P* < 0.001), and (**C**) serum NLRP3 levels (pg/ml) and hepatic CASP1 staining score (r = 0.577, *P* = 0.001) in patients with hepatitis C virus-related liver disease (n = 34).

Immunohistochemical analysis showed that positive immunostaining of NLRP3 and its activation marker CASP1 was detectable as brown granules in the cytoplasm of hepatocytes in liver sections of all patients with HCV-related liver disease (Fig. 2). The final staining score for NLRP3 ranged between 3 and 12 [median (IQR): 7 (3)] with low and high expression were found in 17 (50.0%) patients and 17 (50.0%) patients respectively (Fig. 2A–C). The staining score of CASP1 was classified as score 1 in 8 (23.5%) patients, score 2 in 15 (44.1%) patients and score 3 in 11 (32.4%) patients (Fig. 2D–F). There was a positive correlation between NLRP3 and CASP1 staining scores (r = 0.810, *P* < 0.001) (Fig. 3A).

### Serum NLRP3 levels positively correlate with hepatic expression of NLRP3 and Caspase-1 in patients with HCV-related liver disease

To find out whether NLRP3 levels in serum reflects its liver activity in patients with hepatitis C virus-related liver disease (n = 34), Spearman’s rank correlation was performed and showed that serum NLRP3 levels were positively correlated with hepatic NLRP3 expression (r = 0.749, *P* < 0.001) (Fig. 3B) and CASP1 expression (r = 0.557, *P* = 0.001) (Fig. 3C). Also, serum NLRP3 levels were significantly higher in patients with high NLRP3 expression than in patients with low expression (median (IQR): 1315 (868) pg/ml vs 845 (230) pg/ml respectively, *P* < 0.001).

### No association between serum levels and hepatic expression of NLRP3 and demographic parameters in patients with HCV-related liver disease

Serum levels and hepatic expression of NLRP3 showed no statistically significant correlations with patient’s age (r = 0.227, *P* = 0.116 and r = 0.187, *P* = 0.290 respectively). Also. there were no associations between the increase in NLRP3 levels in serum and liver and sex (male vs female) (median (IQR): 1155 (495) pg/ml vs 920 (408) pg/ml, *P* = 0.754 and 8 (3) vs 6 (5), *P* = 0.479 respectively).

### Statistical correlations between serum levels and hepatic expression of NLRP3, laboratory parameters, the severity of liver dysfunction, and noninvasive fibrosis scores in patients with HCV-related liver disease

Table 2 showed that serum levels and hepatic expression of NLRP3 were positively correlated with serum levels of AST (r = 0.565, *P* < 0.001 and r = 0.432, *P* = 0.011 respectively), ALT (r = 0.574, *P* < 0.001 and r = 0.403, *P* = 0.018 respectively), HCV RNA (r = 0.340, *P* = 0.017 and r = 0.391, *P* = 0.022 respectively), and hsCRP (r = 0.781, *P* < 0.001 and r = 0.542, *P* = 0.001 respectively), APRI (r = 0.526, *P* < 0.001 and r = 0.408, *P* = 0.017 respectively), and FIB-4 (r = 0.460, *P* = 0.001 and r = 0.508, *P* = 0.002 respectively). In patients with cirrhosis, the NLRP3 levels in serum and the liver showed positive correlations only with the ALBI score (r = 0.472, *P* = 0.031 and r = 0.517, *P* = 0.048 respectively) but not with the Child–Pugh score (r = 0.365, *P* = 0.104 and r = 0.384, *P* = 0.157 respectively) or the MELDNa score (r = 0.146, *P* = 0.529 and r = 0.260, *P* = 0.349 respectively).

**Table 2:** Statistical correlations between serum nucleotide-binding and oligomerization domain-like receptor pyrin-domain-containing 3 (NLRP3) levels (pg/ml), hepatic NLRP3 expression, and other parameters in patients with hepatitis C virus (HCV)-related liver disease.

Variables	Serum NLRP3 (pg/ml) (n = 49)		NLRP3 final staining score (n = 34)	
	r value	*P*-value*	r value	*P*-value*
AST (U/L)	0.559	< 0.001	0.443	0.009
ALT (U/L)	0.532	< 0.001	0.394	0.021
GGT (U/L)	0.149	0.308	0.127	0.473
HCV RNA (× 10^3^ IU/ml)	0.340	0.017	0.391	0.022
hsCRP (mg/dl)	0.781	< 0.001	0.542	0.001
Child–Pugh score	0.365	0.104	0.384	0.157
MELDNa score	0.146	0.529	0.260	0.349
ALBI score	0.472	0.031	0.517	0.048
APRI	0.526	< 0.001	0.408	0.017
FIB-4	0.460	0.001	0.508	0.002
Histological activity grade (n = 34)	0.787	< 0.001	0.525	0.001
Fibrosis stage (n = 34)	0.825	< 0.001	0.762	< 0.001
Steatosis grade (n = 34)	0.790	< 0.001	0.734	< 0.001

AST, Aspartate aminotransferase; ALT, Alanine aminotransferase; GGT, Gamma-glutamyl transpeptidase; hsCRP, High-sensitivity C-reactive protein; MELDNa; Model for End-Stage Liver Disease Sodium; ALBI, Albumin-bilirubin; APRI, Aspartate aminotransferase to platelet ratio index; FIB-4, Fibrosis-4 index.

r: Spearman coefficient.

*Statistically significant at *P* ≤ 0.05.

### Increased serum levels and hepatic expression of NLRP3 are associated with significant liver pathology

**Figure 4:**
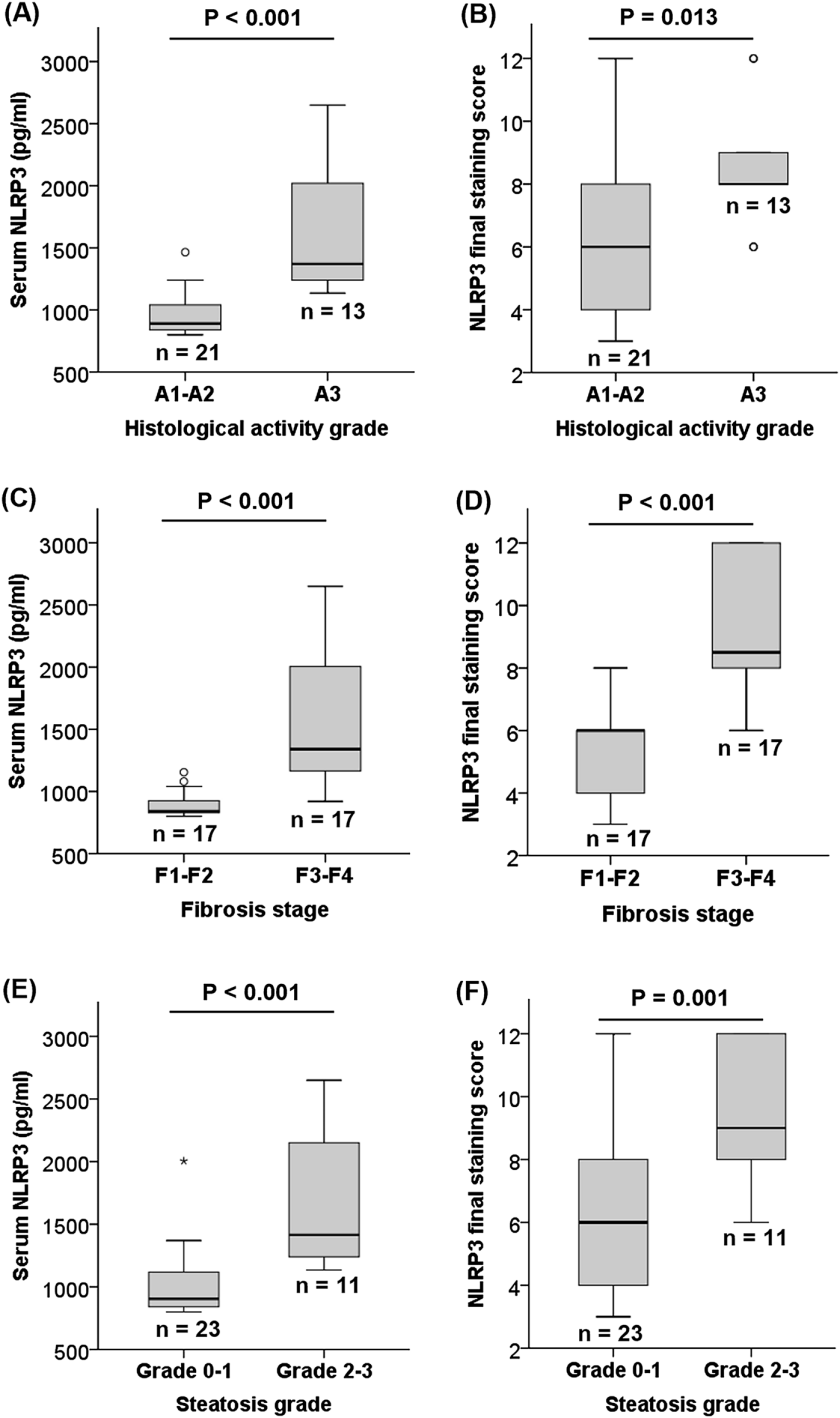
Serum levels (pg/ml) and hepatic expression of nucleotide-binding and oligomerization domain-like receptor pyrin-domain-containing 3 (NLRP3) in: (**A** & **B**) patients with severe necroinflammation (A3) vs patients with mild/moderate necroinflammation (A1-A2) (*P* < 0.001 and *P* = 0.013 respectively), (**C** & **D**) patients with advanced fibrosis/cirrhosis (F3-F4) vs patients with early fibrosis (F1-F2) (*P* < 0.001 for both), and (**E** & **F**) patients with significant steatosis (grade 2–3) vs patients with non-significant steatosis (grade 0–1) (*P* < 0.001 and *P* = 0.001 respectively).

To explore whether increased serum and liver NLRP3 are associated with significant liver pathology, patients with HCV-related liver disease (n = 34) were stratified into two groups according to the severity of liver necroinflammation, fibrosis, and steatosis. Significant increases of serum levels and hepatic expression of NLRP3 were found in patients with severe necroinflammation (A3) compared with patients with mild/moderate necroinflammation (A1-A2) (median (IQR), 1370 (940) vs 890 (223), *P* < 0.001 and 8 (4) vs 6 (4), *P* = 0.013 respectively) (Fig. 4A,B), in patients with advanced fibrosis/cirrhosis (F3-F4) compared with patients with early fibrosis (F1-F2) (median (IQR), 1365 (840) vs 845 (210), *P* < 0.001 and 9 (4) vs 6 (2), *P* < 0.001 respectively) (Fig. 4C,D), and in patients with significant steatosis (grade 2–3) compared with patients with non-significant steatosis (grade 0–1) (median (IQR), 1415 (1040) vs 905 (315), *P* < 0.001 and 9 (4) vs 6 (4), *P* = 0.001 respectively) (Fig. 4E,F). Additionally, positive correlations were found between NLRP3 levels in serum and the liver and histological activity grade (r = 0.787, *P* < 0.001 and r = 0.525, *P* = 0.001 respectively), fibrosis stage (r = 0.825, *P* < 0.001 and r = 0.762, *P* < 0.001 respectively), and steatosis grade (r = 0.790, *P* < 0.001 and r = 0.734, *P* < 0.001 respectively) (Table 2).

### Serum NLRP3 as a potential biomarker for liver necroinflammation, fibrosis, and steatosis

**Figure 5:**
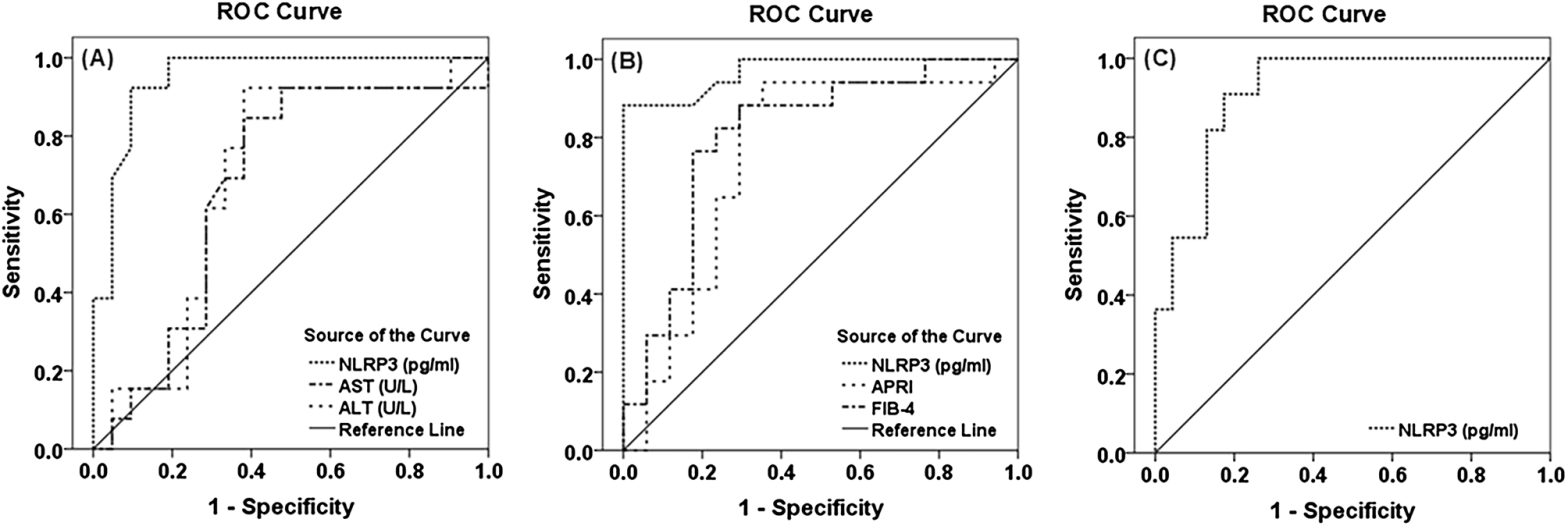
Receiver operating characteristic curve showing the sensitivity and specificity of (**A**) serum nucleotide-binding and oligomerization domain-like receptor pyrin-domain-containing 3 (NLRP3) (pg/ml), serum aspartate aminotransferase (AST) (U/L), and serum alanine aminotransferase (ALT) (U/L) in discriminating severe liver necroinflammation (A3) from mild/moderate liver necroinflammation (A1-A2) (area under the curve (AUC) = 0.951 vs 0.676, *P* = 0.032 and 0.692, *P* = 0.034 respectively), (**B**) serum NLRP3 (pg/ml), aspartate aminotransferase to platelet ratio index (APRI), and Fibrosis-4 index (FIB-4) in discriminating advanced liver fibrosis/cirrhosis (F3-F4) from early liver fibrosis (F1-F2) (AUC = 0.971 vs 0.754, *P* = 0.036 and 0.806, *P* = 0.075 respectively), (**C**) serum NLRP3 (pg/ml) in discriminating significant steatosis (grade 2–3) from non-significant steatosis (grade 0–1) (AUC = 0.917, *P* < 0.001).

The diagnostic performance of serum NLRP3 as a potential biomarker for liver necroinflammation, fibrosis, and steatosis was assessed by plotting the ROC curve (Table 3, Fig. 5). To investigate whether serum NLRP3 could be an additional marker for liver necroinflammation, the sensitivity, specificity, PPV, NPV, and accuracy of serum NLRP3, AST, and ALT in discriminating severe necroinflammation (A3) from mild/moderate necroinflammation (A1-A2) were determined. The comparison between the AUC of ROC curves showed that serum NLRP3 (AUC = 0.951) was superior to serum AST (AUC = 0.676, z = 2.1501, *P* = 0.032) and ALT (AUC = 0.692, z = 2.1186, *P* = 0.034) in distinguishing severe necroinflammation. As regards the extent of liver fibrosis, the sensitivity, specificity, PPV, NPV, and accuracy of serum NLRP3, APRI, and FIB-4 were determined to distinguish advanced fibrosis/cirrhosis (F3-F4) from early fibrosis (F1-F2). The comparison between the AUC of the ROC curves showed that serum NLRP3 (AUC = 0.971) was superior to APRI (AUC = 0.754, z = 2.1004, *P* = 0.036) but was comparable to FIB-4 (AUC = 0.806, z = 1.7829, *P* = 0.075) in the diagnosis of advanced fibrosis/cirrhosis. The diagnostic performance of serum NLRP3 was also high in discriminating significant steatosis (grade 0–1) from non-significant steatosis (grade 2–3) (AUC = 0.917, *P* < 0.001).

**Table 3:** The diagnostic performance of serum nucleotide-binding and oligomerization domain-like receptor pyrin-domain-containing 3 (NLRP3) (pg/ml) in determining the severity of liver necroinflammation, fibrosis, and steatosis.

Variables	AUC	*P* value*	95% CI	Cut-off value	Sensitivity (%)	Specificity (%)	PPV (%)	NPV (%)	Accuracy (%)
Severe necroinflammation (A3) vs mild/moderate necroinflammation (A1-A2)									
NLRP3 (pg/ml)	0.951	< 0.001	0.881–1.000	1162.5	92.3	90.5	95.0	85.7	91.2
AST (U/L)	0.676	0.089	0.485–0.866	74.0	69.2	66.7	56.3	77.8	67.6
ALT (U/L)	0.692	0.095	0.507–0.878	88.0	76.9	66.7	55.9	82.4	70.6
Advanced fibrosis/cirrhosis (F3-F4) vs early fibrosis (F1-F2)									
NLRP3 (pg/ml)	0.971	< 0.001	0.923–1.000	1145	88.2	94.1	93.4	88.9	91.2
APRI	0.754	0.091	0.577–0.932	1.56	76.5	70.6	72.2	75.0	73.5
FIB-4	0.806	0.079	0.652–0.960	1.66	76.5	82.4	81.3	77.8	79.4
Significant steatosis (grade 2–3) vs non-significant steatosis (grade 0–1)									
NLRP3 (pg/ml)	0.917	< 0.001	0.827–1.000	1162.5	90.9	82.6	71.4	95.0	85.3

AST, Aspartate aminotransferase; ALT, Alanine aminotransferase; APRI, Aspartate aminotransferase to platelet ratio index; FIB-4, Fibrosis-4 index; AUC, Area under the curve; CI, Confidence interval; PPV, Positive predictive value; NPV, Negative predictive value.

*Statistically significant at *P* ≤ 0.05.

## Discussion

Accumulating evidence suggests that NLRP3 plays an important role in the pathogenesis of liver diseases^17–24^. The present study demonstrated increased serum NLRP3 levels in patients with HCV-related liver disease in parallel with up-regulation of NLRP3 and its activation marker CASP1 in the liver. In support of this finding, Csak et al.^33^ found elevated expression of NLRP3 and CASP1 at the mRNA level in the livers of HCV-infected patients versus the livers of healthy controls. Also, NLRP3 and active CASP1 were significantly increased in the livers of murine models of immune-mediated hepatitis^23^ and experimental viral fulminant hepatitis^24^ and in HCV-transfected cell lines^34–40^. The close correlation between hepatic expression of NLRP3 and its serum levels may suggest that the liver could be the source of NLRP3 in blood with increased NLRP3 release into the circulation from hepatocytes and non-parenchymal cells and may also indicate that serum NLRP3 could reflect NLRP3 activity in the liver.

Like other RNA viruses, HCV is known as a well-established activator of NLRP3. The present study found an association between HCV viremia and increased NLRP3 levels in serum and the liver. Negash et al.^34^ identified HCV core protein as a virion-specific factor that was necessary and sufficient for NLRP3 activation from macrophages. Also, the HCV glycoprotein E2 was able to potently induce NLRP3 activation in THP-1 macrophages^39^. Ligation of HCV single-stranded RNA to TLR7 in macrophages triggers NLRP3 gene transcription by activating the NF-κB pathway^40^. Moreover, Ramachandran et al.^37^ showed that HCV inhibited ubiquitination of NLRP3 leading to CASP1 activation. Furthermore, HCV activates NLRP3 through the generation of reactive oxygen species^24,33^, potassium efflux^34^, loss of vesicular acidity^41^, and intracellular calcium mobilization^37^. Despite its role in virus defense, persistent activation of NLRP3 with excessive release of pro-inflammatory cytokines and danger signals may promote an inflammatory positive feedback loop that triggers virus replication and persistence^12,13^. Guo et al.^24^ found that *NLRP3 *^*-/-*^ mice and *Caspase-1 *^*-/-*^ mice infected with hepatitis virus strain-3 produced much less viruses as compared to the infected wild-type controls.

The NLRP3-CASP1 pathway plays a central role in the hepatic inflammatory network in chronic liver diseases^9^. The present study showed that serum levels and hepatic expression of NLRP3 increased progressively with increasing severity of liver necroinflammation and were positively correlated with serum aminotransferase levels and histological activity grade (markers of hepatic necroinflammation) in patients with HCV-related liver disease. Consistent with these findings, a previous study showed that the mRNA levels of NLRP3 in the livers of patients with non-alcoholic fatty liver disease (NAFLD), were significantly higher in the livers of patients with non-alcoholic steatohepatitis (NASH) when compared to the livers of patients with non-NASH^19^. Also, Gaul et al.^42^ found that serum and hepatic CASP1 activities were significantly higher in patients with NASH than in patients with simple steatosis or controls and correlated with liver inflammation severity. In addition, *NLRP3* knock-in mice showed severe liver inflammation, with increased infiltration of activated macrophages in a diet-induced NASH model^19^, while *NLRP3*^*-/-*^ mice and *CASP1*^*-/-*^ mice showed decreased histological hepatic injury and serum ALT and AST levels when exposed to liver insults^23,24^. Also, selective pharmacological inhibition of NLRP3 lowered aminotransferases levels and reduced hepatic inflammation in the in vivo NASH models^43,44^ and this effect was associated with a significant reduction in CASP1 activation^44^. CASP1-mediated IL-1β secretion seems to be the crucial mediator of liver inflammation. IL-1β directly induces the synthesis of pro-inflammatory cytokines and chemokines including tumor necrosis factor-α^23,45^ and monocyte chemoattractant protein 1^21^, T helper 17 cell differentiation with the secretion of the proinflammatory cytokine IL-17^23,45^, and recruitment and activation of invariant natural killer T cells^20^.

In the meantime, NLRP3 not only plays a role in hepatic inflammation but also is linked to the systemic inflammatory response^46^ as evidenced by the significant correlation between levels of NLRP3 and hsCRP. Accumulating evidence suggests a mutual relationship between NLRP3 and CRP. A meta-analysis of genome-wide association studies found that the *NLRP3* locus, which encodes the NLRP3, is associated with circulating CRP levels^47^. Meanwhile, CRP can increase the expression of NLRP3 via the FcγRs/NF-κB pathway and up-regulation of reactive oxygen species levels, purinergic receptor signaling, and activation of cysteine proteases^48^. Sendler et al.^46^ found that inhibition of NLRP3 reduced systemic inflammatory response syndrome in mice with severe pancreatitis.

Growing evidence supports a central role of NLRP3 and its downstream pro-inflammatory effectors in liver fibrosis^16,19,43–45^. The present study demonstrated that levels of NLRP3 in serum and the liver were positively correlated with the METAVIR fibrosis stage and showed significant increases with the development of advanced fibrosis/cirrhosis. This result is in agreement with those of previous human and experimental studies. Li et al.^49^ also found that patients with liver fibrosis showed up-regulation of NLRP3 in the liver with alpha-smooth muscle actin and type I collagen expression compared with patients without liver fibrosis suggesting an association between NLRP3 and HSC activation. Moreover, NLRP3 and cleaved CASP1 expression were increased in the livers of patients with cirrhosis compared with the livers of control subjects^18^. In addition, several experimental studies supported the direct role of NLRP3 in liver fibrosis and activation of HSCs with increased profibrotic genes and collagen deposition^19,40,42,45,50,51^. The cytokines and danger signals generated by CASP1 activation bind to receptors located on HSCs resulting in their transdifferentiation through activation of IL-1 type I receptor, c-Jun N-terminal kinase, and activation protein-1 signaling^52^.

Besides, NLRP3 plays a key role in the pathogenesis of hepatic steatosis suggesting a link between chronic inflammation and lipid accumulation in the liver^17,19,23^. The present study showed an association between NLRP3 levels in serum and the liver and the grade of steatosis in HCV-infected patients as also previously demonstrated in patients with NAFLD^17,19,33,53–55^. Also, the expression of NLRP3 and CASP1 in the liver tissues was detected in experimental NAFLD models induced by a high-fat diet compared with the normal diet group^22,33^. Moreover, suppression of NLRP3 activation in the liver reduced lipogenesis, inhibited lipid accumulation in the liver^21,56^, and attenuated hepatic steatosis^22^. The role of NLRP3 in the development of steatosis requires CASP1-mediated inflammation, which promotes triglyceride synthesis in hepatocytes by decreasing lipolytic gene peroxisome proliferator-activated receptor-α transactivation^55,56^, induces tumor necrosis factor-α^23^ and the pro-steatotic chemokine monocyte chemoattractant protein 1 in hepatocytes, and augments TLR4-dependent up-regulation of inflammatory signaling in macrophages^21,45^.

Meanwhile, the present study found that serum NLRP3 could be a potential biomarker for the severity of liver pathology during chronic HCV infection. Serum NLRP3 was superior to serum aminotransferases in determining severe liver necroinflammation and could be an additional inflammatory marker, particularly in patients with normal or slightly raised ALT. Previous investigators found elevated serum levels of NLRP3 in several inflammatory diseases^57–60^. Huang et al.^60^ found that serum NLRP3 level is a useful inflammatory biomarker for identifying high-risk septic patients. Moreover, the present study showed a high diagnostic accuracy of serum NLRP3 in detecting advanced fibrosis/cirrhosis and could be an additional marker with non-invasive fibrosis scores.

## Conclusion

Based on the results of the present study, it can be concluded that NLRP3 plays an important role in liver disease progression during HCV infection through CASP1 activation. Targeting NLRP3 could be a promising therapeutic strategy to limit liver damage, particularly in HCV-infected patients with advanced liver disease who are still at risk for long-term liver-related complications even after sustained virological response. Clinical trials with a large population need to be conducted to validate the use of serum NLRP3 as an additional biomarker for liver inflammation and fibrosis.

## Data Availability

The data presented in this study are available on request from the corresponding author.
